# Midcontinental Native American population dynamics and late Holocene hydroclimate extremes

**DOI:** 10.1038/srep41628

**Published:** 2017-01-31

**Authors:** Broxton W. Bird, Jeremy J. Wilson, William P. Gilhooly III, Byron A. Steinman, Lucas Stamps

**Affiliations:** 1 Department of Earth Sciences, Indiana University-Purdue University, Indianapolis, 46202, USA; 2 Department of Anthropology, Indiana University-Purdue University, Indianapolis, 46202, USA; 3 Large Lakes Observatory and Department of Earth and Environmental Sciences, University of Minnesota Duluth, Duluth, 55812, USA

## Abstract

Climate’s influence on late Pre-Columbian (pre-1492 CE), maize-dependent Native American populations in the midcontinental United States (US) is poorly understood as regional paleoclimate records are sparse and/or provide conflicting perspectives. Here, we reconstruct regional changes in precipitation source and seasonality and local changes in warm-season duration and rainstorm events related to the Pacific North American pattern (PNA) using a 2100-year-long multi-proxy lake-sediment record from the midcontinental US. Wet midcontinental climate reflecting negative PNA-like conditions occurred during the Medieval Climate Anomaly (950–1250 CE) as Native American populations adopted intensive maize agriculture, facilitating population aggregation and the development of urban centers between 1000–1200 CE. Intensifying midcontinental socio-political instability and warfare between 1250–1350 CE corresponded with drier positive PNA-like conditions, culminating in the staggered abandonment of many major Native American river valley settlements and large urban centers between 1350–1450 CE during an especially severe warm-season drought. We hypothesize that this sustained drought interval rendered it difficult to support dense populations and large urban centers in the midcontinental US by destabilizing regional agricultural systems, thereby contributing to the host of socio-political factors that led to population reorganization and migration in the midcontinent and neighboring regions shortly before European contact.

The adoption of intensive agricultural practices allowed North American Pre-Columbian populations (pre-1492 CE) in the southwestern and eastern United States (US) to moderate subsistence shortfalls and thereby sustain larger populations with increasing socio-political complexity^1^,^2^,^3^. One consequence of this agricultural strategy, however, was an inherent susceptibility to climate change and water resource availability, especially in the absence of water retention and/or diversion infrastructure^2^,^4^,^5^,^6^,^7^. In the southwest, interrelationships between population dynamics, agricultural productivity, and climate are illustrated by the expansion of agriculturally based Native American populations during pluvial (wet) conditions prior to 1000 CE and their subsequent collapse during the Medieval Climate Anomaly (MCA) between 1000 and 1300 CE in response to a series of multi-decadal droughts that severely limited agricultural yields (Fig. 1) 7,8,9.

A similar climate-agriculture-population dynamic has been suggested to explain the arc of midcontinental Native American populations associated with Mississippian and related archaeological cultures of the Ohio River Valley (i.e., Fort Ancient and Monongahela) between 1000–1450 CE^10^,^11^,^12^. These populations collectively expanded between 1000–1200 CE, developing large villages and socio-cultural/religious centers with higher population densities likely sustained by the adoption of intensive maze agriculture by 1000 CE^12^,^13^,^14^. Maize was added to a diet including previously domesticated starchy and oily seeds of the Eastern Agricultural Complex and is believed to have been grown in both floodplain and upland contexts^15^. In the Mississippi and Ohio River drainages, hundreds of Mississippian villages and regional polities of varying sizes and configurations were developed during this time, including Cahokia, Kincaid and Angel, which were among the largest (Fig. 1). At their height, population densities in larger floodplains of the midcontinental US approached 27 individuals per km^2^ 13, similar to that of modern-day Missouri^16^. At the same time, Fort Ancient and Monongahela polities also emerged further upstream along the Ohio River, relying on similar subsistence patterns to sustain increasing population aggregation in smaller villages^17^. This late Pre-Columbian population reorganization, was soon followed by a period of increased warfare and militarization between 1250–1350 CE that culminated in the widespread and somewhat synchronous decline of population densities and urban centers across the central Mississippi and lower Ohio River valleys. The first indications of discord at Mississippian settlements occurred at Cahokia and within the American Bottom, where some upland urban centers and smaller settlements alike were abandoned starting around 1150 CE during a severe drought, culminating in the whole of the central Mississippi valley being depopulated by 1350 CE. Between ~1350–1450 CE, the Illinois and lower Ohio River valleys experienced a similar, progressive abandonment of larger centers with most Mississippian populations shifting to the lower Mississippi River valley and interior southeast by 1500 CE^18^. Population densities would remain low in this so-called midcontinental “Vacant Quarter” throughout the European contact phase, not reaching former levels until after the 1900s^19^,^20^.

While climate has been suggested as a component of late Pre-Columbian Native American settlement patterns and population dynamics in the midcontinental US^20^,^21^,^22^,^23^, paleoclimate records with the length, temporal resolution, and proxy sensitivity required to identify the potential effects of past climate change on agricultural potential are scarce and provide conflicting climatic information. Early work relying on a synthesis of tree-ring based drought reconstructions and archaeological records suggests that Cahokia developed during wet conditions prior to 1150 CE and that a switch to more frequent drought after this time contributed to its collapse^7^,^21^. In contrast, Munoz *et al*.^23^ identified suspected flood deposits in two lakes near Cahokia and suggested that increased regional precipitation and flooding after 1200 CE contributed to the collapse of Cahokia and other Mississippian city centers. Though these contrasting climatic scenarios for the late Pre-Columbian period are not mutually exclusive, they do illustrate the need for paleoclimate records that capture geographically broad climate processes capable of influencing populations spanning a large region.

To this end, we developed a high-resolution multi-proxy lake sediment record from Martin Lake, Indiana, USA (41.56°, −85.38°, 274 m ASL; Fig. 1). This record captures both local and synoptic-scale (≥1000 km horizontal scale) climate variability related to the Pacific North American teleconnection (PNA). We use this dataset to investigate the potential linkages between abrupt climate change, the intensification of maize agriculture, and late Pre-Columbian population dynamics. Specifically, sub-decadally resolved down-core measurements of the oxygen isotopic composition of authigenic carbonates (δ^18^O_cal_) were used to reconstruct changes in the source and seasonality of precipitation, which are in turn correlated to PNA-like synoptic-scale atmospheric circulation patterns that affect climate across the Ohio River valley region. Local variations in the duration and intensity of the warm-season and associated rainfall events, both of which are also correlated to the mean state of the PNA for the central Mississippi and Ohio River valleys, were reconstructed using the carbon isotopic composition of authigenic carbonates (δ^13^C_cal_) and variations in the abundance of lithic material (terrestrially derived inorganic sediment), respectively. By linking synoptic-scale PNA variability with regional and local climatic changes, our multi-proxy approach provides an integrated perspective on midcontinental hydroclimate variability during the last 2100 years, lending new insight into the large-scale climate patterns associated with Pre-Colombian Native American cultural changes.

## Results and Discussion

### Study Site

**Figure 1:**
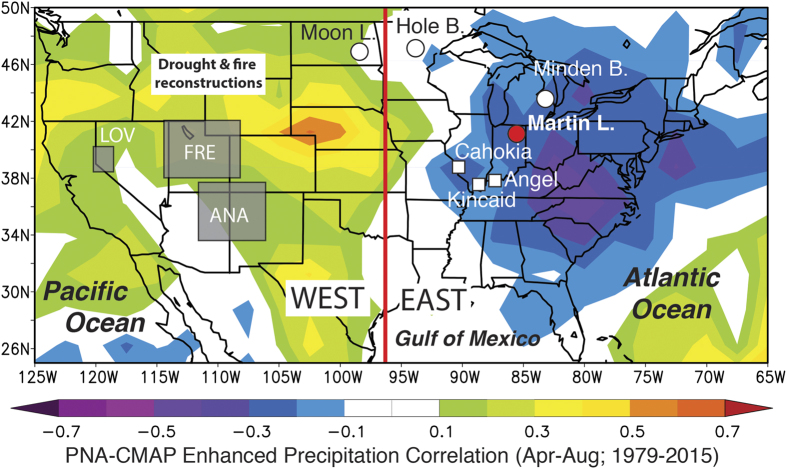
Map of the continental US showing the location of Martin Lake (red circle), other paleoclimate records (white circles) and major midcontinental Native American sites (white squares). The white text rectangle indicates that drought and fire reconstructions are from the broader western US^5^,^44^. Gray boxes in the western US indicate locations of Anasazi (ANA), Fremont (FRE) and Lovelock (LOV) cultures that collapsed between 1000 and 1300 CE^7^. Colored shaded regions indicate correlations between growing season (Apr-Aug) CMAP-enhanced precipitation and the PNA index. Image provided by the NOAA/ESRL Physical Sciences Division, Boulder Colorado (http://www.esrl.noaa.gov/psd/).

Martin Lake is a 17 meter-deep monomictic head-water kettle lake in northeastern Indiana (Figs 1 and S1). The lake is fed by ephemeral streams that merge into a single inflow on Martin Lake’s east side with additional hydrologic contributions from near surface groundwater (i.e., base flow)^24^. Year-round outflow drains to Olin Lake through a modified natural outlet on the lake’s western shore. Regional precipitation (910 mm yr^−1^) results in approximately 1.18 × 10^7^ m^3^ of precipitation over the lake’s 12.86-km^2^ catchment, which is more than ten times Martin Lake’s volume (1.11 × 10^6^ m^3^). This produces hydrologically open conditions and a short mean residence time of 103 days^24^. Despite its open hydrology, Martin Lake maintains persistent thermal stratification during the warm-season from April through November, which results in bottom water anoxia from March through December (Fig. S1)^25^. Also during the warm-season, biogenically mediated calcium carbonate is precipitated in the lake’s upper water column, which under anoxic conditions results in the accumulation of rhythmically repeating mm-scale couplets comprised of discrete detrital/organic matter and authigenic calcite laminae (Fig. S2).

### Precipitation, Martin Lake Water and Authigenic Calcite δ^18^O

**Figure 2:**
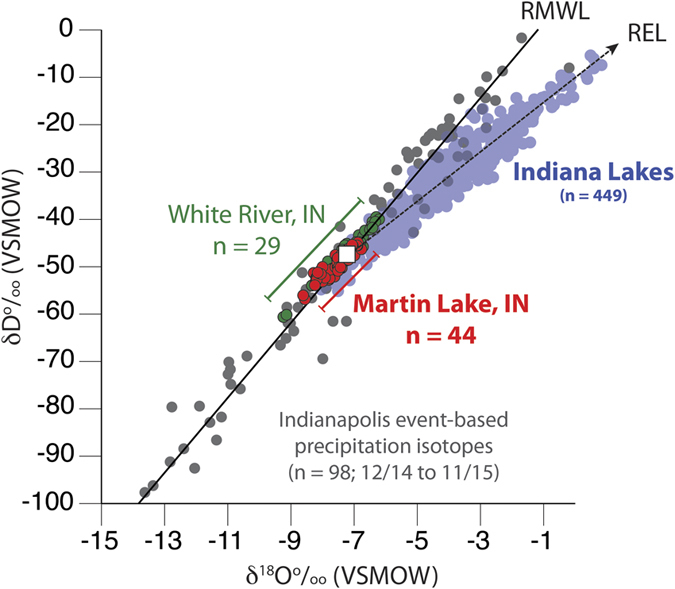
Hydrogen and oxygen isotope results for Indianapolis, IN, precipitation events (gray circles), Martin Lake, IN (red circles), Indiana lakes (light blue circles), and the White River, IN (green circles). The white box indicates the intersection between the REL and RMWL that defines average annual δ^18^O_precip_ for Indiana.

Event-based precipitation δ^18^O measurements from Indianapolis, IN (n = 98) collected between December 2014 and November 2015 define a regional meteoric water line (RMWL) with a slope of 7.9 and y-intercept of 9.4, consistent with the global meteoric water line (GMWL; Fig. 2) 26. The unweighted annual δ^18^O_precip_ value of −8.3‰ is similar to modeled annual average δ^18^O_precip_ for the region (−8.1‰)^27^ and to the seasonally weighted annual δ^18^O_precip_ value (−8.0‰) determined from back trajectory analysis of the event-based δ^18^O_precip_ measurements (discussed below). Surface and water column samples from Martin Lake (n = 44; average δ^18^O_lw_ = −7.6‰) plot along the RMWL and are therefore consistent with meteoric waters (Fig. 2 and Table S1). Were Martin Lake influenced by evaporation, its surface waters would plot along the regional evaporation line (REL), which is defined by 449 surface water samples collected from lakes across Indiana between 2010 and 2015. Average Martin Lake δ^18^O_lw_ and δD_lw_ are instead within 0.2‰ and 1.4‰ of their respective REL-RMWL intercept values (Table S1). The correspondence between Martin Lake surface water isotope values and the REL-RMWL intercept indicates that Martin Lake water reflects the regional average isotopic composition of precipitation for Indiana, which includes a large portion of the lower Ohio River valley.

Isotopic measurements of modern calcite from littoral vegetation at Martin Lake (−8.4‰) are identical within error to predicted δ^18^O_cal_ (−8.5‰), given an average warm-season near-surface temperature of 18 °C^25^ and average δ^18^O_lw_ of −7.6‰. This indicates that authigenic calcite precipitates in equilibrium with Martin Lake surface water and thus that down-core measurements of δ^18^O_cal_ can be used as a reliable proxy for past changes in regional δ^18^O_precip_.

### Modern δ^18^O_precip_ Controls

**Figure 3:**
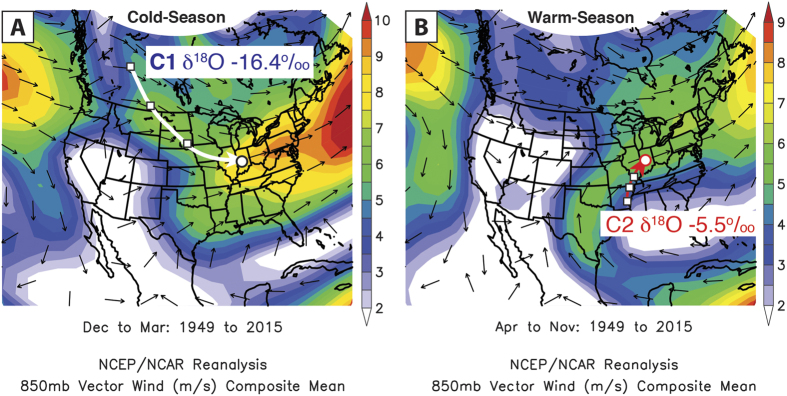
Composite mean maps of (**A**) cold-season (Dec-Mar) and (**B**) warm-season (Apr-Nov) 850 hPa vector winds between 1949–2015 from the NCEP-NCAR reanalysis database. Arrows are wind directions with colored shading indicating velocity (m/s). Also plotted are mean back trajectories for cluster 1 (thick white line in **A**) and cluster 2 (thick red line in **B**) of the event-based Indianapolis, IN, precipitation samples. White squares are spaced at 24 hr intervals. White circles indicate the starting point from which back trajectories were calculated. The mean back trajectories are in good agreement with their respective seasonal atmospheric circulation patterns since 1949, indicating the precipitation back trajectories are representative of the modern climatological mean. Images provided by the NOAA/ESRL Physical Sciences Division, Boulder Colorado (http://www.esrl.noaa.gov/psd/).

Back trajectory and cluster analysis of the event-based precipitation samples were conducted with the US National Oceanic and Atmospheric Administration (NOAA) Hybrid Single-Particle Lagrangian Integrated Trajectories (HYSPLIT) model using the NOAA Global Data Assimilation System data^28^. Consistent with modern midcontinental climatology^29^, the results show that warm-season (Apr-Nov) rainfall accounts for 74.5% of mean annual precipitation and occurs mainly as discrete rainstorms when southerly mean atmospheric flow advects high δ^18^O_precip_ (−5.5‰) from the Gulf of Mexico into the midcontinent (Fig. 3 and Table S1). Cold-season (Dec-Mar) precipitation accounts for 25.5% of annual totals and is sourced from Pacific and Arctic regions with characteristically low δ^18^O_precip_ (−16.4‰), resulting in a moisture source δ^18^O difference of 10.9‰. The average δ^18^O_precip_, weighted to reflect warm and cold-season contributions, equals −8.0‰. This is consistent with the REL-RMWL intercept, modeled δ^18^O_precip_ and average Martin Lake δ^18^O_lw_, further supporting that Martin Lake δ^18^O_lw_ reflects regional climate.

Changes in the proportion of northerly and southerly moisture sources strongly affect δ^18^O_precip_ across the midcontinental US and are linked to the PNA^30^, the leading mode of continental US climate variability on seasonal and longer timescales^31^. During negative (−) PNA phases, the atmospheric circulation pattern increases midcontinental precipitation originating from ^18^O-enriched southerly moisture sources^30^,^31^,^32^, and reduces the amount of precipitation in the southwestern US. Conversely, during positive (+) PNA phases, the frequency of ^18^O-depleted northwesterly air mass incursions into the midcontinental US increases. As a result, the precipitation amount is reduced and δ^18^O_precip_ values are lower in the Midwest during +PNA phases, while portions of the western US become wetter. On decadal and longer timescales, the PNA is modulated by the Pacific Decadal Oscillation (PDO)^30^,^33^, which is the multi-decadal, North Pacific expression of the El Niño-Southern Oscillation^34^, such that +PDO events are typically concurrent with a +PNA phase, and vice versa.

Relevant to this investigation, the strongest PNA-precipitation correlations in North America are located over Indiana and the central Mississippi and lower Ohio River valleys, with correlations (r) as high as −0.71 (Fig. S4)^32^. Similar precipitation-PNA correlations (based on data from 1949 and 2015 CE) exist on seasonal (April-August) and annual timescales, demonstrating a consistent long-term PNA-hydroclimate relationship in the midcontinental US (Figs 1 and S4). Paleoclimate records from the midcontinental US that can be linked to PNA variability are therefore useful for investigating regional hydroclimate patterns associated with this teleconnection and their relationship with Pre-Columbian Native American cultural changes.

### Late Holocene Midcontinental Hydroclimate Variability

**Figure 4:**
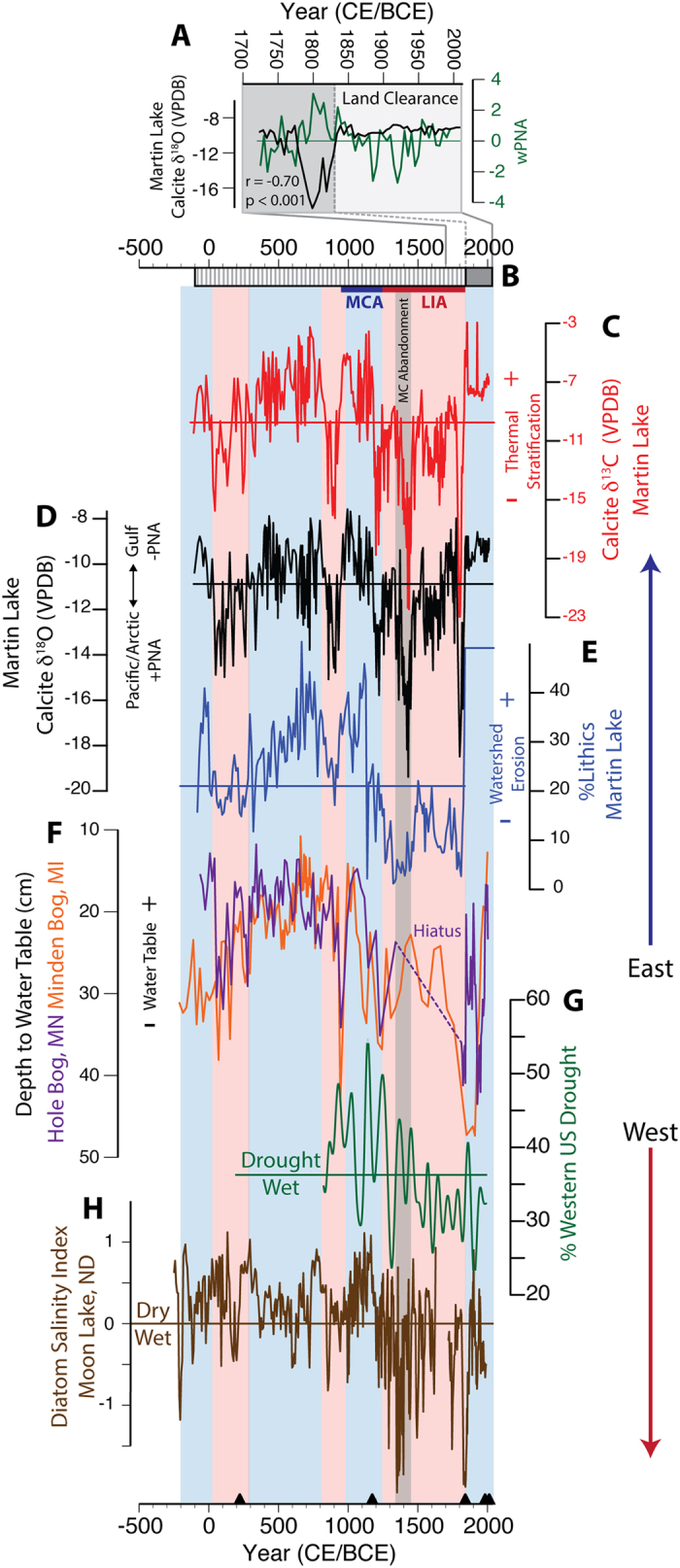
(**A**) Comparison of Martin Lake δ^18^O_cal_ with reconstructed winter PNA variability since 1725 CE. Dark gray shading indicates undisturbed laminated sediments. Light gray shading designates mottled sediments reflecting watershed land clearance. (**B**) Simplified Martin Lake stratigraphic profile. Lined sections represent laminated sediments, gray shading indicates mottled sediments. Martin Lake results for (**C**) δ^13^C_cal_, (**D**) δ^18^O_cal,_ and (**E**) % lithics (y-axis truncated at 60%) plotted against (**F**) water level reconstructions at Hole Bog, MN, (purple) & Minden Bog, MI, (orange), (**G**) tree-ring based western US drought percentage (30-year low-pass filtered) and (**H**) the Moon Lake diatom-inferred salinity index. Shaded vertical boxes define midcontinental pluvials (blue), droughts (red) and the midcontinental (MC) abandonment between 1350–1450 CE (gray). Colored horizontal lines indicate time series averages. Black triangles designate ^14^C dates. Dashed purple line in F demarcates the Hole Bog LIA drought hiatus.

Continuous down-core δ^18^O_cal_ measurements with a 5-year resolution from Martin Lake show considerable variability and a 12.5‰ range (Fig. 4). Temperature is unlikely to have contributed substantially to δ^18^O_cal_ variability because the combined influence of temperature-controlled isotopic fractionation of liquid water in the air mass (+0.59‰ °C^−1^)^26^ and calcite from lake water (−0.22‰ °C^−1^)^35^ would require an unrealistically large change in temperatures (e.g., a change of approximately 34 °C is needed to explain the full range). Instead, we contend that the 12.5‰ range in Martin Lake δ^18^O_cal_ reflects mean state changes in the predominance of northerly and southerly moisture sources, which have an isotopic difference of 10.9‰ (Fig. 1). Because the PNA exerts strong control on regional midcontinental δ^18^O_precip_^30^, we interpret periods with high δ^18^O_cal_ to reflect sustained −PNA-like phases at Martin Lake, as well as over the central Mississippi and lower Ohio River valleys, leading to high δ^18^O_precip_ and δ^18^O_cal_ values. Conversely, +PNA-like phases promoted cold-season-like northerly atmospheric flow over the midcontinental US, resulting in low δ^18^O_precip_ and δ^18^O_cal_ values. This interpretation is supported by the strong correlation between Martin Lake δ^18^O_cal_ and a reconstruction of the winter PNA index (wPNA) from 1725 to 1840 CE based on 12 tree-ring records from the continental US and Alaska^36^. The Martin Lake-wPNA δ^18^O correlation (r = −0.70, p < 0.001; Fig. 4) is similar to those of the modern PNA-precipitation relationship^32^, thus further supporting the use of down-core Martin Lake δ^18^O_cal_ measurements as an indicator of synoptic-scale PNA-like variability. The Martin Lake record is strongly impacted by regional land clearance after the 1840 s^37^, with increased deposition from watershed erosion in response to deforestation and greater catchment discharge^38^. Therefore, the weakened post-1840 CE wPNA-Martin Lake δ^18^O_cal_ correlation is interpreted to reflect a change in watershed hydrology as a result of large-scale land clearance, rather than a disconnect between δ^18^O_precip_ and the PNA. Nonetheless, high average δ^18^O_cal_ at Martin after 1840 CE is in line with a generally negative PNA mean-state during the last two centuries^36^, and together with the strong pre-1840 correlation, supports interpreting down-core δ^18^O_cal_ in terms of mean state changes in δ^18^O_precip_ related to PNA-like variability.

Changes in the relative contributions of moisture sources were estimated using a two-component δ^18^O_precip_ mixing model with end-member δ^18^O_precip_ values representing northwesterly (−16.4‰; +PNA) and southerly (−5.5‰; −PNA) moisture sources that were scaled to match past δ^18^O_cal_ variability during specific time intervals (Tables S1 and S2). High δ^18^O_cal_ between 950 and 1190 CE suggests that −PNA-like conditions prevailed at Martin Lake and over the central Mississippi and lower Ohio River valleys during the MCA, with southerly warm-season rainfall accounting for approximately 68% of annual precipitation during early population expansion among the Mississippian, Fort Ancient and Monongahela cultures (Fig. 4). Archaeological data show infrequent signs of warfare-related trauma and minimal construction of city center fortifications during this time, suggesting relatively low levels of conflict^11^,^13^. Decreasing δ^18^O_cal_ between 1190–1250 CE marks a shift to +PNA-like conditions in the midcontinental US that persisted throughout the LIA. This time period was characterized by approximately equal distributions of warm and cold-season precipitation and coincides with a rapid rise in fortification construction around Mississippian cities. Subsequently, an especially pronounced δ^18^O_cal_ minimum between 1400–1470 CE marks the strongest +PNA-like phase of the last 2100 years, contemporaneous with the final abandonment of midcontinental Mississippian cities in the central Mississippi and lower Ohio River valleys. During this timeframe, midcontinental precipitation was largely from northerly cold-season sources (79%) and summer precipitation was substantially reduced. By 1500 CE, these same and related Mississippian populations were concentrated in the lower Mississippi River valley and interior of the Southeast where they encountered the Spanish during the 16^th^ century^11^,^20^. Modern seasonal precipitation distributions were established after 1830 CE, as climate transitioned toward the current warm period (CWP; 1900 CE to present).

The strong covariance between Martin Lake δ^18^O_cal_ and δ^13^C_cal_ data (r = 0.81; p < 0.001; Fig. 4) supports the inferences on Pre-Columbian precipitation seasonality derived from the δ^18^O_cal_ record alone. Prolonged warm-season thermal-stratification in midcontinental lakes similar to Martin Lake increases the sequestration of ^13^C-depleted organic matter in anoxic bottom water, leaving surface water enriched in ^13^C and resulting in high δ^13^C_cal_^39^. Because midcontinental warm-season precipitation is dominated by southerly moisture sources with high δ^18^O values, both δ^13^C_cal_ and δ^18^O_cal_ increase during extended warm-seasons^39^. The opposite occurs when climate is colder and moisture is predominantly sourced from northerly regions.

**Figure 5:**
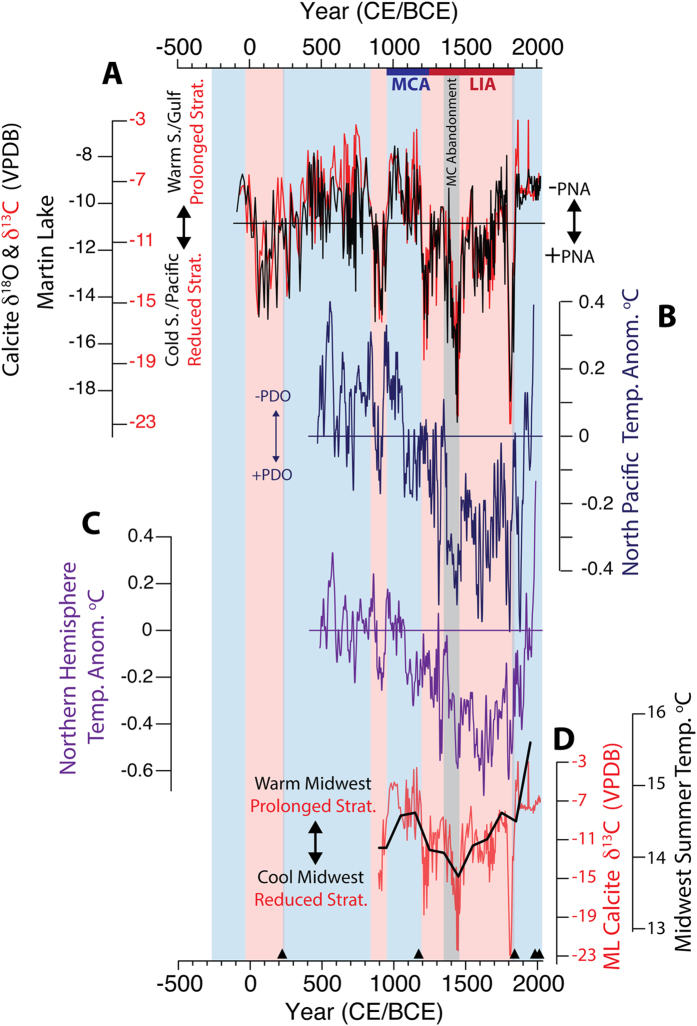
PNA variability inferred from Martin Lake (**A**) δ^18^O_cal_ (black) and δ^13^C_cal_ (red) compared with (**B**) PDO and (**C**) Northern Hemisphere temperatures during the last 1500 years. (**D**) Martin Lake δ^13^C_cal_ is compared with a multi-site pollen-based Midwestern summer temperature reconstruction, showing that thermal stratification closely follows regional temperature variability. Shaded boxes, horizontal lines and black triangles as defined in Fig. 4.

Prolonged stratification during the early expansion of late Pre-Columbian populations (950–1200 CE) indicates extended warmth that was associated with southerly sourced moisture. This is consistent with warmer Midwestern summers^40^ and above-average Northern Hemisphere temperatures^41^ during the MCA, suggesting the δ^13^C_cal_ values reflect not only warmer temperatures at Martin Lake, but regionally across the midcontinental US (Fig. 5). In contrast, reduced stratification during the LIA when moisture was sourced from northerly regions indicates shorter warm-seasons, especially between 1400–1470 CE. These trends were associated with cooler Northern Hemisphere and Midwestern summer temperatures, supporting regionally cooler midcontinental temperatures during the LIA with peak cooling between 1400–1470 CE^40^ when the Vacant Quarter was established in the central Mississippi and lower Ohio River valleys (Fig. 5).

The concentration of lithic material (the inorganic clastic sediment fraction) in Martin Lake sediments (Fig. 4) provides a record of terrestrial erosion resulting from warm-season precipitation events critical for the non-irrigated maize agriculture practiced by the Mississippians and related late Pre-Columbian populations. Sediment delivery to Martin Lake occurs predominantly during the warm-season when the lake is ice-free and abundant rainfall from southerly derived rainstorms mobilizes watershed sediment. Greater warm-season precipitation, i.e., more frequent rainstorm events, would therefore result in greater sediment delivery to Martin Lake; drier conditions would have the opposite effect. This interpretation is consistent with the co-occurrence of high lithic concentrations during the MCA when δ^18^O_cal_ and δ^13^C_cal_ values were also elevated, indicating enhanced warm-season rainfall and watershed erosion during warm, extended −PNA-like conditions. Conversely, low lithic abundances coincide with low δ^18^O_cal_ and δ^13^C_cal_ during the LIA, indicating reduced warm-season rainfall when cold-season conditions predominated under a more persistent +PNA-like mean state.

Although the lithics results are reflective of local precipitation over the Martin Lake watershed, they indicate changes in the frequency of southerly derived rainstorms, which follow trajectories that track over the central Mississippi and lower Ohio River valleys (Fig. 3). Changes in the frequency of these rainstorms therefore would impact areas along warm-season storm trajectories, including the central Mississippi and lower Ohio River valleys. This is reflected in the modern relationship between midcontinental rainfall and the phase of the PNA (i.e., reduced during +PNA phases and increased during −PNA phases; Fig. 1)^32^. The Martin Lake lithics results therefore suggest that regions which today experience increased warm-season precipitation during −PNA phases were wetter (i.e., more frequent southerly derived warm-season rainstorms) during the MCA, but drier during the LIA when +PNA-like conditions predominated. Minimum lithics concentrations between 1350–1450 CE during the strongest +PNA-like phase of the last 2100 years, indicate an especially pronounced PNA-related warm-season drought during the Mississippian abandonment of the midcontinent.

The occurrence of synchronous synoptic- and local-scale PNA-like hydroclimate variability in the midcontinental US suggested by the Martin Lake δ^18^O_cal_, δ^13^C_cal_ and lithic data are supported by other regional paleoclimate records including amoeba testate water-table reconstructions from Minden (Michigan) and Hole Bog, MN (Mississippi headwaters; Fig. 4)^42^. These records, from locations that are today similarly affected by PNA variability, demonstrate generally elevated water tables during the MCA and CWP (−PNA). Conversely, water tables were lower at both sites during the LIA between 1250 and 1800 CE (+PNA) with a depositional hiatus at Hole Bog during this time indicating a dry climate in the upper Mississippi watershed. These results are in good agreement with the Martin Lake record and indicate that midcontinental sites separated by as much as ~1000 km were similarly and synchronously affected by PNA-like hydroclimate variability during the MCA, LIA and CWP. This supports the synoptic-scale nature of the hydroclimate variability captured by the Martin Lake record and its extrapolation to adjacent regions in the central Mississippi and lower Ohio River valleys (~500 km from Martin Lake), where midcontinental Mississippians and related Pre-Columbian cultures were centered.

The strong evidence for regional midcontinental drought after 1250 CE contradicts assertions of a wetter LIA, and more specifically that suspected increases in flooding at Cahokia after 1200 CE were indicative of larger-scale flooding that contributed to the broader Mississippian abandonment of the midcontinent^23^. Cahokia’s location below the confluence of the Missouri and Mississippi Rivers complicates interpretations of flood records from this location, in that attribution to a specific river drainage is not possible, thus preventing any firm conclusion regarding the nature of climate change and the putative flood packages recorded in the American Bottom^23^. We therefore suggest that if increased flooding did indeed occur at Cahokia after 1200 CE, it reflects increased discharge from the Missouri River, for which the majority of the drainage basin is located in the western US. This assertion is consistent with records that indicate greater warm-season precipitation across much of the western US during the LIA, including the Missouri River drainage^5^,^43^,^44^, while midcontinental conditions were drier.

The PNA-like mean state changes in midcontinental hydroclimate variability identified in the Martin Lake and regional paleoclimate records are supported on a continental-scale by tree-ring drought reconstructions^5^, wild-fire histories^44^, and lake salinity^43^ records from the western US (Fig. 4). Regionally coherent western warm-season drought and increased fire occurrence during the MCA, when midcontinental records indicate pluvial conditions, mirrors modern east-west antiphased PNA-hydroclimate correlations (Fig. 1). The opposite conditions occurred during the LIA, consistent with a reversal in the US hydroclimate gradient. These paleoclimate inferences support the idea of a persistent east-west dipole pattern in warm-season precipitation across the continental US that closely resembles modern warm-season PNA-precipitation relationships and, furthermore, that PNA-like hydroclimate variability has affected the distribution of pluvial and drought conditions on multi-decadal to centennial timescales during the last 2100 years.

Consistent with Pacific atmosphere-ocean modulation of the PNA and North American droughts, the PNA-like hydroclimate anomalies indicated by the Martin Lake and regional paleoclimate data largely track reconstructed PDO variability during the last 1500 years (r = 0.51, p < 0.01 for 15-yr moving averages of each time series; Fig. 5)^4^,^33^,^41^. Negative PNA-like mean states correspond to generally negative PDO-like conditions (the MCA) and vice versa (the LIA). The most prominent +PNA events recorded at Martin Lake (850–950, 1400–1470 and 1800–1820 CE) correspond to +PDO phases. Other δ^18^O_cal_ minima (+PNA) also correspond with +PDO events, but with differences in the δ^18^O_cal_ response magnitude (Fig. 5).

### Hydroclimate and Midcontinental Native American Cultural Trends

**Figure 6:**
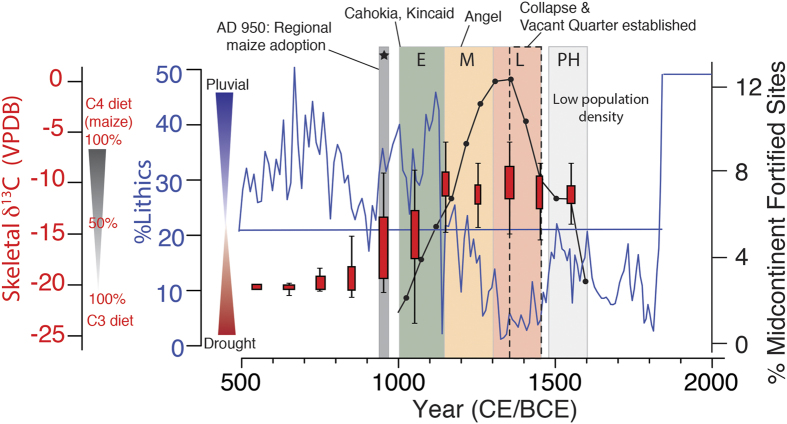
Martin Lake lithics as an indicator of warm-season rainfall (blue line) compared with human skeletal δ^13^C of midcontinental Pre-Columbian populations that reflects diet (red box and whisker plots), the percentage of fortified Mississippian sites as an indicator of conflict (black line and ovals) and notable events during the rise and fall of midcontinental human populations. Shaded boxes represent the adoption of maize agriculture (gray box with star) and the early (E; green), middle (M; orange) and late (L; red) Mississippian periods, and the post-historic period (PH; light gray). The dashed box marks the 1350–1450 CE midcontinental abandonment. Human skeletal δ^13^C values after 1450 CE are from sites located outside of the Vacant Quarter.

Late Pre-Columbian population growth between 1000 and 1200 CE was associated with extended warm-season conditions during the MCA that were accompanied by above average precipitation derived from southerly moisture sources (Fig. 6). These climatic conditions would have been favorable for the adoption of intensive swidden agriculture, maize in particular, which enabled population growth and sustained emerging population centers. This scenario is consistent with modeling results^21^ that demonstrate that early agriculture would have been positively impacted by increased precipitation during late Pre-Columbian population expansion, when climatic conditions were favorable to growth and aggregation. An increased proportion of juveniles occurred in large skeletal assemblages from eastern North America dating to this timeframe^45^, suggesting elevated crude birth rates, consistent with more abundant and reliable food sources that positively impacted female reproductive ecology.

The widespread adoption of maize agriculture by midcontinental Native American populations during the early MCA is confirmed by a newly compiled regional database of human skeletal δ^13^C comprised of more than 1300 individuals from across the eastern US that reflects dietary changes during the last 2300 years (Figs 6 and S5 and Table S3). An abrupt increase in human skeletal δ^13^C from background values of −20.3‰ (350 BCE to 950 CE) to −15.9‰ between 950–1050 CE at sites across the Midwest and into Pennsylvania reflects a regional dietary change from one relying exclusively on C3 plants to one where 50% or more of protein was provided by C4 plants (i.e., maize)^45^. This supports the hypothesis that expanding populations, aggregation and social complexity were fueled by the rapid and widespread adoption of intensive maize agriculture between 950 and 1050 CE made possible by an opportune climate^12^,^13^.

The signatures of warfare and conflict, including skeletal trauma and active defensive features (i.e., palisades with bastions), increased markedly after 1150 CE across the midcontinent (Fig. 6)^11^. Consistent with the modeled climate-agriculture-population response^12^, these trends mirrored declining warm-season precipitation during the transition to the LIA, the very same period when maize consumption peaked among Native American populations (i.e., human skeletal δ^13^C values from −10.3 to −10.1‰ between 1150–1450 CE). As LIA aridity became the new mean climatic state, farmsteads and hamlets gave way to larger, aggregated villages in many major river valleys, suggesting that safety superseded other necessities and constrained resource catchment areas^18^. In the central Illinois River valley, for example, several fortified villages show evidence for full-scale conflagration between 1200 and 1350 CE, alluding to frequent raiding and competition for declining resources. Furthermore, paleodemographic analyses of skeletal samples from this region demonstrate an increasing age-specific risk of death that was particularly severe among reproductive-age Mississippian females, likely because pathogen loads and disease transmission were higher in “compact village life” conditions, which slowly eroded the reproductive ecology and sustainability of these villages^46^. The progressive collapse of Mississippian populations between 1350–1450 CE coincides with exceptionally severe warm-season drought and extended cold-seasons. Shorter and drier growing seasons during this time would have significantly reduced agricultural yields, thereby contributing to intensified resource-related conflict that ultimately undermined the socio-political fabric and population dynamics of midcontinental Mississippian societies^10^,^11^,^12^,^13^,^22^.

Native American populations in the upper Ohio River Valley associated with the Monongahela and Fort Ancient cultures, as well as Mississippian groups in the southeastern and eastern US, persisted after depopulation in the lower Ohio and central Mississippi River valleys. While it is not clear exactly how these populations in the upper Ohio River Valley persevered through the LIA-associated drought episodes, the spatial and temporal patterning of settlements indicate that they were less ubiquitous after 1500 CE, with significant buffer zones between communities^18^,^20^. There are also indications that Fort Ancient villages shifted from year-round to seasonal occupation during the LIA, with an increased emphasis on hunting, including bison, to potentially cope with food production shortfalls and reduced maize consumption^17^,^47^. These smaller, constrained populations may have persevered due to 1) local climatic conditions, 2) a reduced population size that could be supported by floodplain agriculture alone, or 3) local socio-political autonomy that was more resilient and responsive to drought-induced resource stress. In the southeastern and eastern US (e.g., New York, the Ontario Peninsula, and regions along the east coast and lower drainages of the Delaware and Susquehanna Rivers), the persistence and even thriving, of Native American populations during and following the midcontinental abandonment^20^ is consistent with a decreasing PNA influence on climate with increasing distance from the Ohio River Valley^32^, as illustrated by the attenuated variability in paleoclimate records spanning the last millennium as compared with the Martin Lake results^48^,^49^,^50^.

## Conclusions

The emergence and expansion of Mississippian and related populations of the midcontinental US after 1000 CE was associated with the adoption of intensive maize agriculture under favorable climatic conditions with extended warm-seasons and above average midcontinental rainfall during the MCA when a −PNA-like climate phase predominated. In contrast, Mississippian socio-political systems show evidence for increased stress and conflict during the transition to the LIA as the PNA switched to a more positive phase and midcontinental summer precipitation was significantly reduced. Following this pattern, the ultimate abandonment of urban centers across the lower Ohio and central Mississippi River valleys between 1350–1450 CE was associated with sustained drought. These climatic-archaeological associations support the idea that although socio-political dynamics were likely an important factor in midcontinental Native American cultural changes during the last millennium, background climatic conditions set the stage for cultural change and the movement of peoples by either favoring or discouraging the stability of large agriculturally based midcontinental Native American populations and associated socio-political systems.

## Methods

### Core collection and processing

Ten surface and long sediment cores were retrieved from Martin Lake’s profundal zone between May and October 2013 using a modified Livingstone piston corer, a modified surface piston corer and freeze corer^51^,^52^. Core segments were overlapped by 30 to 50 cm to ensure complete recovery. All cores were transported to the IUPUI Paleoclimatology and Sedimentology Laboratory where they were stored at 4 °C. After being split into archive and work halves, cores were imaged using a GeoTek Multi Sensor Core Logger under cross-polarized LED lighting.

### Sediment characterization

X-ray diffraction (XRD) and scanning electron microscope (SEM) analysis was conducted on six samples from core D13 Drive 1: 4–4.5, 54–54.5; Drive 2: 54.0–54.5; Drive 3: 34.0–34.5, 84.0–84.5; and Drive 4: 64.0–64.5 at the IUPUI Integrated Nanosystems Development Institute. Powdered XRD analysis was conducted using a Siemens (now Bruker) D5000. A JEOL 7800 F field emission SEM was used to characterize carbonate crystal morphology.

### Age control

Accelerator mass spectrometry (AMS) radiocarbon (^14^C) ages were determined for eight samples comprised of charcoal fragments or terrestrial plant material at the University of California Irvine (UCI) Keck Carbon Cycle AMS Facility (Table S4). Samples were collected using a binocular microscope, mechanically cleaned and pretreated with an acid-base-acid wash (1 N HCl and 1 N NaOH) following UCI protocols. AMS ^14^C ages were calibrated to common era and before common era years (CE/BCE) using the online application CALIB^53^. The online application CALIBomb was used for sample UCIAMS# 132273 because it contained excess ^14^C^54^. The age-depth model was created by fitting a 4^th^ order polynomial to radiocarbon ages below 16 cm and a linear age model on two age control points above this depth (one ^14^C ages and the modern surface water interface of 2013 CE; Fig. S6).

### Carbonate δ^18^O and δ^13^C analysis

Sediments were sampled continuously at 0.5 cm intervals for calcite oxygen and carbon isotope analysis (δ^18^O_cal_ and δ^13^C_cal_). Calcite was isolated following standard proceedures^55^ and measured at the IUPUI Stable Isotope Biogeochemistry Laboratory on a MAT 252 isotope ratio mass spectrometer coupled with a GasBench II system after reaction with 100% phosphoric acid at 70 °C. Results are reported in delta notation and normalized relative to Vienna Pee Dee Belemnite (VPDB) using the international standards NBS-18, NBS-19 and IAEA CO-9 with 0.1‰ precision for δ^18^O and δ^13^C.

### Percent Lithics

Approximately 1.0 g of wet sediment was collected at 1 cm intervals for grain size analysis. Following Gray *et al*.^56^, samples were dried at 60 °C for 24 hr, weighed, and then soaked for 24 hr in a 25 mL aliquot of 30% H_2_O_2_ at room temperature to begin the process of removing organic material. Three to five additional 20 mL aliquots of 30% H_2_O_2_ were applied at 65 °C, followed by a rinse with DI water. Biogenic silica was removed by soaking samples in a 20 mL 1 N NaOH solution for 8 hr at 60 °C while carbonates were digested with a 1 N HCL solution for 1 hr at room temperature. Following these treatments, samples were freeze-dried and weighed again to calculate the abundance of lithic and mineral fragments (% lithics).

### Meteoric and surface water isotope measurements

Precipitation events >0.02 mm between 12/1/2014 and 11/30/2015 (n = 98) were collected in Indianapolis, IN, using an evaporation free system modified from^57^. Samples were collected in Indianapolis instead of at Martin Lake because the daily collection schedule required supervision that was not possible at the lake site. The annual average isotopic differences in δ^18^O_precip_ and δD_precip_ between Indianapolis and Martin Lake are small, however (0.5 and 6‰, respectively), supporting the regional application of our results from Indianapolis. The strong agreement between Martin Lake δ^18^O_lw_ and δD_lw_ and Indiana δ^18^O_precip_ and δD_precip_ based on the former’s correspondence with the RMWL and REL additionally supports the regional similarity of the δ^18^O_precip_ signal captured in Indianapolis and at Martin Lake.

River water samples for δ^18^O and δD analysis were collected at least monthly from the White River, IN, (n = 29) between 11/14/2014 and 11/30/2015. The White River runs through Indianapolis, IN, and drains a watershed measuring 3265 km^2^. Water samples from natural lakes, reservoirs and impoundments across Indiana collected by the Indiana Clean Lakes Project at Indiana University between May 2010 and August 2015 were also analyzed for δ^18^O and δD (n = 449).

Water column samples for δ^18^O_lw_ and δD_lw_ analysis were collected from Martin Lake in May, September, and October 2013. Simultaneous water column measurements of temperature (°C) and dissolved oxygen (DO mg/L) were obtained at 1 m intervals using a Hydrolab MS5. Water samples from May 2013 were collected at 3 m intervals from 0 to 16 m while those in September and October were collected at 1 m intervals from 0 to 16 m. Surface water samples from Martin Lake were additionally collected from June-September 2015 and in January 2016.

All water samples were analyzed at IUPUI for δ^18^O and δD using a Picarro L2130-i Analyzer coupled to an autosampler and high-precision water vaporizer unit. Measurements were corrected for memory and drift following the methodology of Gröning *et al*.^58^. Final values were corrected to the VSMOW scale using calibrated standards from Los Gatos. Precision for δ^18^O and δD is 0.1 and 0.6‰, respectively.

### Precipitation back trajectory analysis

Back trajectory analysis of the Indianapolis, IN, precipitation events (n = 98) was conducted with the US National Oceanic and Atmospheric Administration’s Hybrid Single-Particle Lagrangian Integrated Trajectories (HYSPLIT) model using Global Data Assimilation System (GADS) data. Back trajectories were initiated at 1500 m above ground level and calculated for the 72 hours prior to the precipitation event. Cluster analysis of the back trajectories was conducted with the HYSPLIT software.

### Estimating past moisture source mixing

Variations in the contribution of northerly and southerly moisture sources to mean annual δ^18^O_precip_ were calculated for specific periods of interest using a simple binary mixing model:


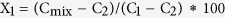


where X_1_ is the proportion of end-member 1 in annual average δ^18^O_precip_ (in percent), C_1_ is the δ^18^O_precip_ value of end-member 1, C_2_ is the δ^18^O_precip_ value of end-member 2, and C_mix_ is the resulting annual average δ^18^O_precip_ (Tables S1 and S2).

End-member δ^18^O_precip_ values were set using the precipitation event cluster analysis with warm-season δ^18^O_precip_ representing end-member 1 (C_1_ = −5.5‰) and cold-season δ^18^O_precip_ representing end-member 2 (C_2_ = −16.4‰). For C_mix_, δ^18^O_lw_ was used as a proxy for δ^18^O_precip_ given the close modern relationship between these variables. Average δ^18^O_lw_ was back calculated from δ^18^O_cal_ using an average warm-season calcite precipitation temperature of 18 °C^35^. Changes in the temperature of calcite precipitation by ±2 °C equate to ±0.5‰ for δ^18^O_lw_ and ±5% for proportional changes in seasonal moisture source contributions to estimated mean annual δ^18^O_precip_.

### Human skeletal carbon isotopes

Proxy δ^13^C data on the incorporation of maize into the diet and subsistence systems of indigenous midcontinental populations from the middle to late Holocene were compiled from 28 sources, representing 1,303 individuals geographically spanning the Missouri, Mississippi, and Illinois River valley eastward through the Ohio River drainage system and southern Great Lakes (references in supplemental materials; Table S3). This dataset was supplemented by ongoing isotopic and geochemical research on the ~1725 BP skeletal assemblage from Mann site 12Po2, a Middle Woodland archaeological site in southwestern Indiana. Where available, direct radiometric assays on bone collagen were recalibrated in OxCal 4.2. In instances where direct radiocarbon dates for interments were not obtained, the published chronometric dates for the archaeological site were recalibrated to develop a summed probability of site occupation. The median date for the individual or site was then used to calculate the age before present (BP; present = 1950 CE). One-hundred year, time-transgressive boxplots from 2300 to 300 BP were subsequently created to plot against the hydroclimate proxy measures and archaeological record of midcontinental fortifications.

**Data Availability.** All original data is archived with the NOAA Paleoclimatology Database (https://www.ncdc.noaa.gov/paleo/study/21061).

## Additional Information

**How to cite this article**: Bird, B. W. *et al*. Midcontinental Native American population dynamics and late Holocene hydroclimate extremes. *Sci. Rep.* **7**, 41628; doi: 10.1038/srep41628 (2017).

**Publisher's note:** Springer Nature remains neutral with regard to jurisdictional claims in published maps and institutional affiliations.

## Supplementary Material

Supplementary Information
