# Supplementary material for: Midcontinental Native American population dynamics and late Holocene hydroclimate extremes
Source: Sci Rep. 2017 Jan 31;7:41628. doi: 10.1038/srep41628 (PMC5282493; doi:10.1038/srep41628)
Supplement: Supplementary Information [file srep41628-s1.pdf]

Title: Midcontinental Native American population dynamics and late Holocene hydroclimate extremes

Authors: [1]Broxton W. Bird, [2]Jeremy J. Wilson, [1]William P. Gilhooly III, [3]Byron A. Steinman and [1]Lucas Stamps

Affiliations:
[1]Department of Earth Sciences, Indiana University-Purdue University, Indianapolis, 46202, USA.
[2]Department of Anthropology, Indiana University-Purdue University, Indianapolis, 46202, USA.
[3]Large Lakes Observatory and Department of Earth and Environmental Sciences, University of Minnesota Duluth, Duluth, 55812, USA.

Corresponding Author: Broxton W. Bird, Department of Earth Sciences, Indiana University-Purdue University, 723 West Michigan St., SL118, Indianapolis, IN 46202; (317) 274-7468; bwbird@iupui.edu

Key Words: North American paleoclimate, Little Ice Age, Medieval Climate Anomaly, Pacific North American mode, Mississippians

Supplemental Text

Human skeletal carbon isotope references 1-27 are located in the reference section after the figures.

Supplemental Figures

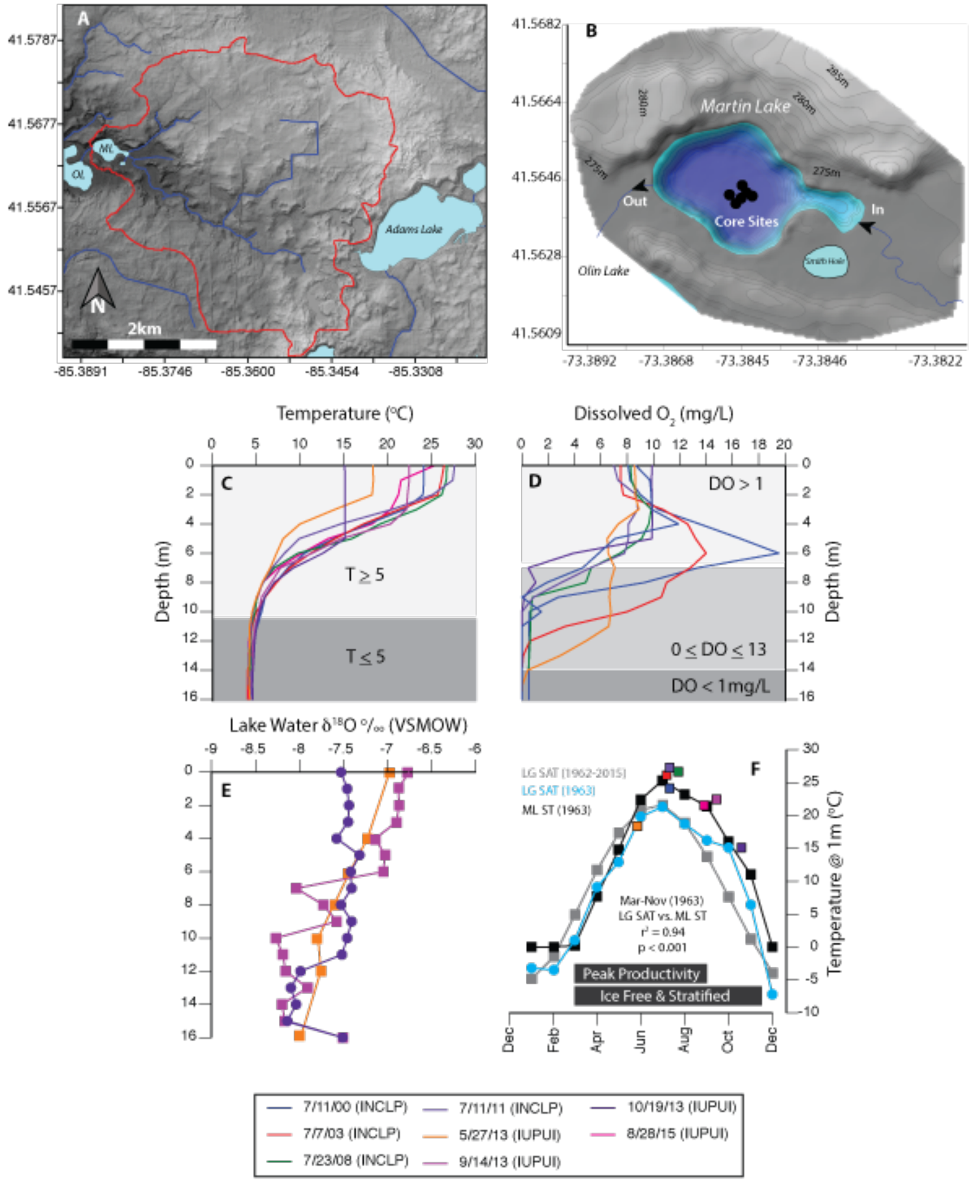

Figure S1 (A) LiDAR digital elevation model of the Martin Lake (ML) watershed (red line; 12.86 km^2). Streams are shown in dark blue. Those in the Martin Lake watershed are ephemeral and have an average channel slope of 0.5%. The watershed boundaries and stream slopes were determined using the USGS on-line StreamStats program (http://streamstats.usgs.gov). Water bodies are light blue (OL = Olin Lake). (B) Bathymetric map of Martin Lake and proximal watershed showing the location of its inflow, outflow and core sites (black circles). Martin Lake water column profiles of (C) temperature (ºC) and (D) dissolved oxygen (mg/L) measured between 7/11/2000 and 8/28/2015. Measurements are color coded by date and investigator (i.e., Indiana Clean Lakes Program or Indiana University-Purdue University, Indianapolis). These profiles show persistent warm-season thermal stratification with bottom water anoxia below 14 m and seasonal anoxia extending up to 7 m. Gray boxes represent water column regions based on temperature and dissolved oxygen concentrations. (E) Water column $\delta^{18}O$ profiles. (F) Average monthly surface air temperatures from La Grange, IN, (LG SAT) for 1963 (blue) and from 1962-

2015 (gray) are compared with Martin Lake surface temperatures (ML ST) from 1963[28]. 1963 surface air and lake surface temperatures are significantly correlated with subsequent measurements (colored squares) showing similar seasonal patterns. Black bars indicate the period during which Martin Lake is ice free and stratified and when primary productivity peaks. Maps in (a) and (b) were created using Goldern Software's Surfer 12 mapping program (http://www.goldensoftware.com/products/surfer).

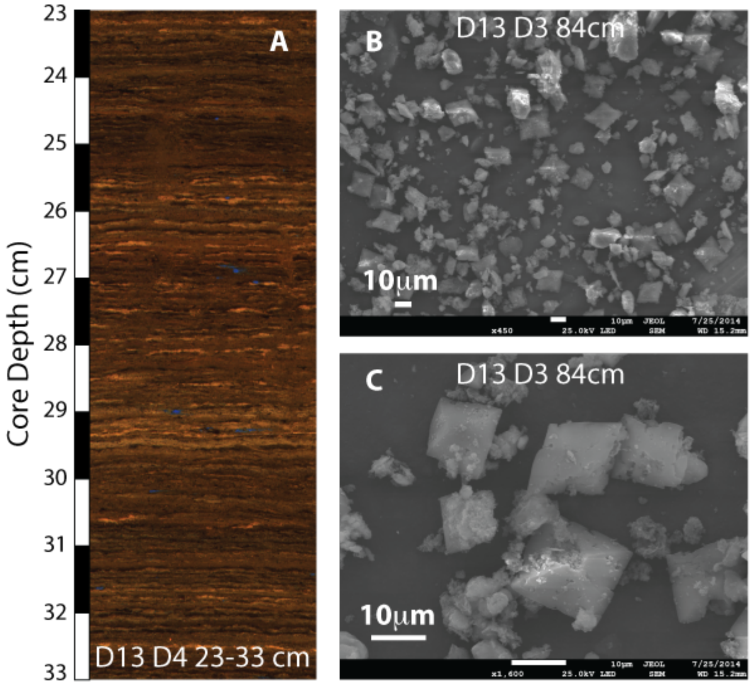

Figure S2 (A) GEOTEK image of a representative stratigraphic section from Martin Lake core D13 drive 4 between 23-33 cm showing the laminated nature of the sediment. Light laminae are comprised of calcite while dark layers are comprised of organic material and lithics. Blue specks in the image are oxidized vivianite. (B) SEM image of calcite crystals from Martin Lake core D13 drive 3 at 84 cm. (C) Enlarged SEM image of D13 drive 3 at 84 cm showing the euhedral structure of calcite crystals.

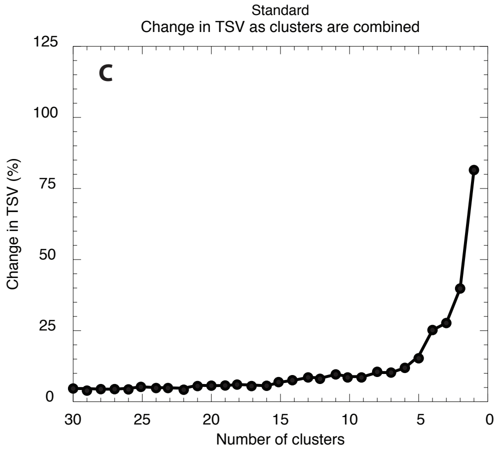

Figure S3 Percent change in total spatial variance (TSV) captured as the number of clusters was consecutively reduced by one in the HYSPLIT cluster analysis of the event-based Indianapolis precipitation isotope data.

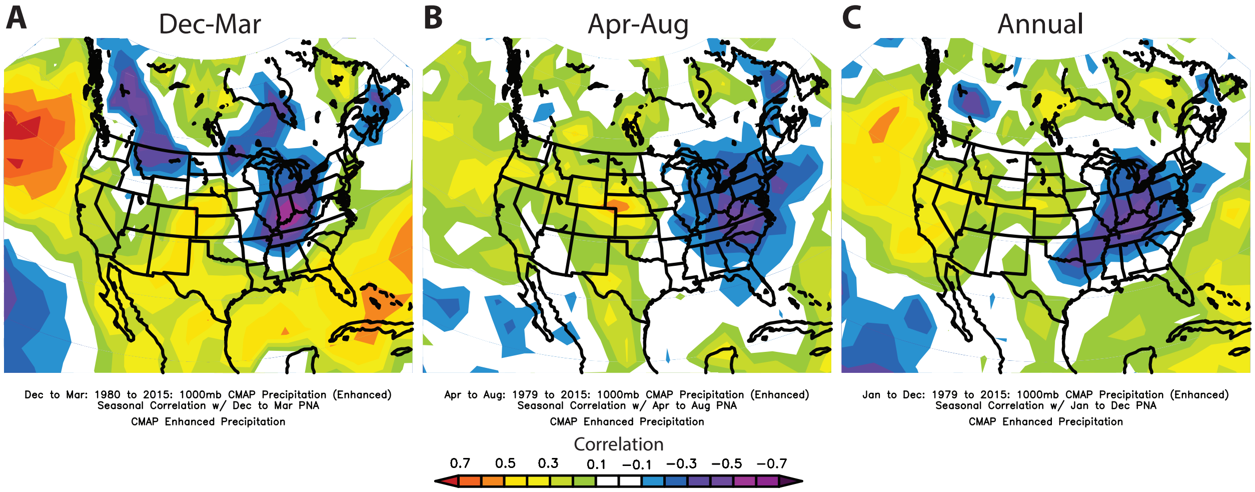

Figure S4 Seasonal correlation maps between (A) Dec-Mar (B) Apr-Aug, and (C) Jan-Dec precipitation (CMAP-enhanced) and the PNA index. The PNA-precipitation correlation is consistently negative in the eastern US during the warm- and cold-seasons and throughout the year. The western US PNA-precipitation correlation is positive during the growing season from April to August and for the annual average. Winter (Dec-Mar) PNA-precipitation correlations, however, reverse for parts of the Pacific northwest, creating a north-south dipole in addition the general east-west dipole. Images provided by the NOAA/ESRL Physical Sciences Division, Boulder Colorado from their Web site (http://www.esrl.noaa.gov/psd/).

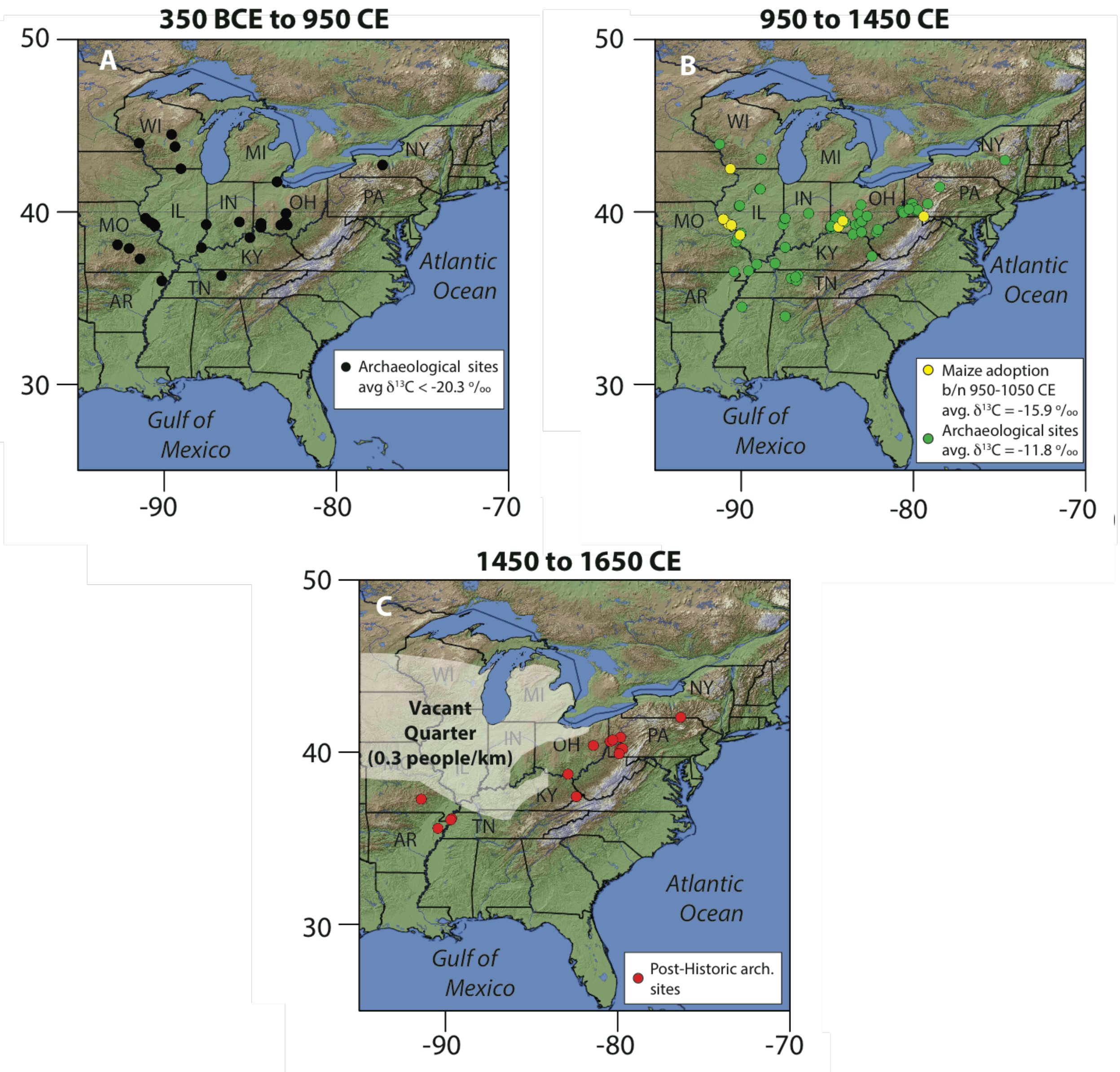

Figure S5 Maps of the eastern half of the US showing (A) the distribution of Pre-Columbian archaeological sites occupied at least intermittently between 350 BCE and 950 CE for which human skeletal $\delta^{13}C$ was measured (black circles). Average $\delta^{13}C$ these sites is consistent with a hunter-gatherer diet lacking signficant contributions of maize protines (-20.3‰). (B) Yellow circles show Pre-Columbian archaeological sites with the first evidence for the adoption of maize agriculture between 950 and 1050 CE (yellow circles) as indicated by average human skeletal $\delta^{13}C$ values consistent with maize comprising at least 50% of diets (approximatley -15‰)[29]. Green circles show Pre-Columbian sites occupied at various points between 1050 and 1450 CE with average $\delta^{13}C$ values of -11.8‰, indicating wide spread intensive maize agriculture and consumption. (C) Post-historic archaeoloigcal sites with evidence of occupation after the establishment of the Vacant Quarter (white shaded region; after Milner and Chaplin[19]). Maps were created using Golden Software's Surfer 12 mapping program (http://www.goldensoftware.com/products/surfer).

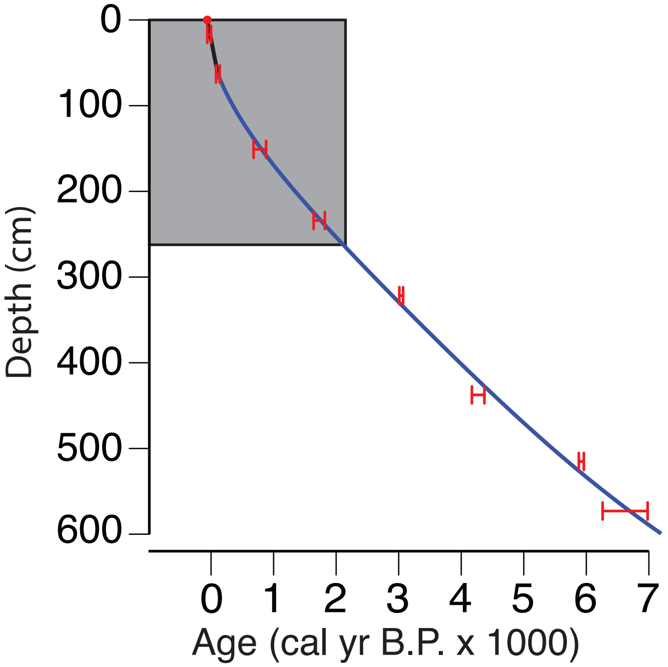

Figure S6 Calibrated Martin Lake AMS ^{14}C ages from Table S4 vs. their respective composite core depths (cm) with the modern sediment-water interface marked with a black circle. Two-sigma age ranges are shown with the horizontal red line. The blue line shows the 4th order polynomial age-depth model up to 1980 CE while the black line shows the three point linear age model after 1980 CE. The gray box indicates the portion of the core that spans the interval of this study.

Supplemental References

Human skeletal $\delta^{13}C$ references: #1-27

Figure References: #28-29

1 Baerreis, D. A. & Bender, M. M. The Outlet Site (47 Da 3): Some dating problems and a reevaluation of the presence of corn in the diet of middle and late Woodland peoples in Wisconsin. *Midcontinental Journal of Archaeology* **9**, 143-154 (1984).

2 Bender, M. M., Baerreis, D. A. & Steventon, R. L. Further light on carbon isotopes and Hopewell agriculture. *Am. Antiq.* **46**, 346-353 (1981).

3 Broida, M. *An estimate of the percents of maize in the diets of two Kentucky Fort Ancient villages*. 68-82 (Kentucky Heritage Commission, 1984).

4 Buikstra, J. E. *et al.* Diet, demography, and the development of horticulture. *Emergent Horticultural Economies of the Eastern Woodlands, Occasional Paper* **7**, 67-86 (1987).

5 Buikstra, J. E. & Milner, G. R. Isotopic and archaeological interpretations of diet in the Central Mississippi Valley. *Journal of Archaeological Science* **18**, 319-329 (1991).

6 Buikstra, J. E., Rose, J. C. & Milner, G. R. *A carbon isotopic perspective on dietary variation in late prehistoric western Illinois*. (Office of the State Archaeologist, University of Iowa, 1994).

7 Bumsted, M. P. *Human variation: $\delta^{13}C$ in adult bone collagen and the relation to diet in isochronous C4 (maize) archaeological diet* PhD thesis, University of Massachusetts Amherst, (1984).

8 Bush, L. L. *Boundary conditions: Macrobotanical remains and the Oliver phase of central Indiana, AD 1200-1450*. (University of Alabama Press, 2004).

9 Cook, R. A. & Schurr, M. R. Eating between the lines: Mississippian migration and stable carbon isotope variation in Fort Ancient populations. *American Anthropologist* **111**, 344-359 (2009).

10 Emerson, T. E., Hedman, K. M. & Simon, M. L. Marginal horticulturalists or maize agriculturalists? Archaeobotanical, paleopathological, and isotopic evidence relating to Langford Tradition maize consumption. *Midcontinental Journal of Archaeology* **30**, 67-118 (2005).

11 Farrow, D. C. A study of Monongahela subsistence patterns based on mass spectrometric analysis. *Midcontinental Journal of Archaeology* **1**, 153-179 (1986).

12 Greenlee, D. M. *Accounting for subsistence variation among maize farmers in Ohio Valley prehistory* Ph.D. thesis, University of Washington, (2002).

13 Hedman, K. M. Late Cahokian subsistence and health: Stable isotope and dental evidence. *Southeastern Archaeology* **25**, 258-274 (2006).

14 Hedman, K., Hargrave, E. A. & Ambrose, S. H. Late Mississippian diet in the American Bottom: stable isotope analyses of bone collagen and apatite. *Midcontinental Journal of Archaeology* **27**, 237-271 (2002).

15 McCall, A. E. *The relationship of stable isotopes to Late Woodland and Fort ancient agriculture, mobility, and paleopathologies at the Turpin Site* M.Sc. thesis, University of Cincinnati, (2013).

16 Rose, F. Intra-community variation in diet during the adoption of a new staple crop in the Eastern Woodlands. *Am. Antiq.* **73**, 413-439 (2008).

17 Schurr, M. R. Isotopic and mortuary variability in a Middle Mississippian population. *Am. Antiq.* **57**, 300-320 (1992).

18 Schurr, M. R. & Powell, M. L. The role of changing childhood diets in the prehistoric evolution of food production: an isotopic assessment. *Am. J. Phys. Anthropol.* **126**, 278-294 (2005).
19 Schurr, M. R. & Redmond, B. G. Stable isotope analysis of incipient maize horticulturists from the Gard Island 2 site. *Midcontinental Journal of Archaeology* **57**, 69-84 (1991).
20 Schurr, M. R. & Schoeninger, M. J. Associations between agricultural intensification and social complexity: an example from the prehistoric Ohio Valley. *Journal of Anthropological Archaeology* **14**, 315-399 (1995).
21 Strange, M. *The effect of pathology on the stable isotopes of carbon and nitrogen: implications for dietary reconstruction* MA thesis, Binghamton University, SUNY, (2006).
22 Tubbs, R. M. *Ethnic identity and diet in the central Illinois River valley* Ph.D. thesis, Michigan State University, (2013).
23 Vogel, J. C. & Van Der Merwe, N. J. Isotopic evidence for early maize cultivation in New York State. *Am. Antiq.* **42**, 238-242 (1977).
24 Van der Merwe, N. J. & Vogel, J. C. ^{13}C content of human collagen as a measure of prehistoric diet in woodland North America. *Nature* **276**, 815-816 (1978).
25 Vradenburg, J. A. Skeletal analysis of the Tremaine Site. *Manuscript on file at the Museum Archaeology Program of the State Historical Society of Wisconsin, Madison* (1993).
26 Ambrose, S. H., Buikstra, J. & Krueger, H. W. Status and gender differences in diet at Mound 72, Cahokia, revealed by isotopic analysis of bone. *Journal of Anthropological Archaeology* **22**, 217-226 (2003).
27 Wells, J. J. *The Vincennes phase: Mississippians and ethnic plurality in the Wabash drainage of Indiana and Illinois* Ph.D. thesis, Indiana University, (2008).
28 Wetzel, R. Productivity investigations of interconnected marl lakes (I). The eight lakes of the Oliver and Walters Chains, northeastern Indiana. *Hydrobiological Studies* **3**, 91-143 (1973).
29 Boutton, T., Klein, P., Lynott, M., Price, J. & Tieszen, L. in *Stable isotopes in nutrition* 191-204 (American Chemical Society, 1984).

Supplemental Tables

Variable	$\delta^{18}O$‰	δD ‰	$\Delta\delta^{18}O$‰ relative to *Martin L.*
Martin Lake, La Grange, IN,<br>*recent surface waters*<br>n = 11<br>6/15 – 9/15	-7.6	-50.8	
Martin Lake, La Grange, IN, long-term avg.<br>n = 41<br>0-16 m avg.<br>7/11 – 1/16	-7.6	-50.5	0.0
White River, Indianapolis, IN<br>n = 29<br>11/23/14 to 11/14/15	-7.1	-46.4	+0.5
Annual mo. avg. precipitation<br>Indianapolis, IN<br>n = 98 events<br>12/01/14 to 11/30/15	-8.3	-56.3	-0.7
LMWL – LEL Intercept	-7.4	-49.1	+0.2
Cluster 1<br>n = 25<br>25.5% of total<br>72.0% from Dec – Mar<br>Source: Pacific/Arctic	-13.7	-110.6	
Cluster 2<br>n = 73<br>74.5% of total<br>80.8% from Apr – Nov<br>Source: Gulf of Mexico/Atlantic	-6.7	-43.5	
Cluster 1 & 2 weighted annual avg.<br>25.5% C1 $\delta^{18}O$ & δD<br>74.5% C2 $\delta^{18}O$ & δD	-8.3	-59.2	-0.7
Cluster 1 cold-season<br>n = 18<br>Dec – Mar	-16.4	-126.2	
Cluster 2 warm-season<br>n = 59<br>Apr – Nov	-5.5	-33.8	
Cluster 1 & 2 weighted seasonal avg.<br>76.6% warm season Apr – Nov<br>23.4% cold season Dec – Mar	-8.0	-53.4	-0.4

Table S1

Average isotopic composition of modern water samples from Martin Lake, the White River, IN, annual monthly precipitation and the LMWL-LEL intercept. Also shown are isotopic values for annual and seasonal back trajectory clusters 1 and 2 of Indianapolis, IN, precipitation events. The right column expresses the ‰ difference between variables and Martin Lake $\delta^{18}O_{lw}$.

	1950 to present	1830 to present	1400 to 1470 CE	1250 to 1830 CE	950 to 1250 CE	870 to 950 CE	400 to 830 CE	±
**±Average $d^{18}O_{cal}$**	-9.3	-9.5	-15	-12.1	-9.9	-12.5	-10.1	
**$d^{18}O_{lw}$ @ 18° C***	-8.4	-8.6	-13.9	-11	-8.7	-11.4	-9	
**Warm-season %**	73%	72%	23%	50%	71%	46%	68%	
**Cold-season %**	27%	28%	77%	50%	29%	54%	32%	
**$d^{18}O_{lw}$ @ 16° C**	-8.9	-9.1	-14.4	-11.5	-9.2	-11.9	-9.5	-0.5‰
**Warm-season %**	69%	67%	18%	45%	66%	41%	63%	-5%
**Cold-season %**	31%	33%	82%	55%	34%	59%	37%	-5%
**$d^{18}O_{lw}$ @ 20° C**	-7.9	-8.1	-13.4	-10.5	-8.2	-10.9	-8.5	+0.5‰
**Warm-season %**	78%	76%	28%	54%	75%	50%	72%	+5%
**Cold-season %**	22%	24%	72%	46%	25%	50%	28%	+5%

*Temperature from Wetzel[28]

Table S2

Back calculations of $\delta^{18}O_{lw}$ based on $\delta^{18}O_{cal}$ assuming calcite precipitation at modern average surface temperature (18º C) and ± 2º C (16º and 20º C). End member $\delta^{18}O_{precip}$ values for warm-season and cold-season sources are based on seasonal $\delta^{18}O_{precip}$ values from clusters 1 and 2 shown in Table 1.

Before Present	Mean	Median	N	Std. Deviation	Std. Error of Mean
2300 BP	-20.6938	-20.6850	16	.08973	.02243
2200 BP	-19.3500	-19.3500	2	.63640	.45000
2000 BP	-20.0500	-20.0500	2	.49497	.35000
1900 BP	-20.6625	-20.6500	24	.35973	.07343
1800 BP	-21.4085	-21.5300	26	.75402	.14788
1700 BP	-21.1722	-21.0000	36	1.08222	.18037
1600 BP	-20.3591	-20.7500	22	2.45602	.52363
1500 BP	-20.5707	-20.7000	14	.44515	.11897
1400 BP	-19.9100	-20.1700	25	1.12436	.22487
1300 BP	-19.4484	-20.3000	31	2.32864	.41824
1200 BP	-19.7024	-20.2000	21	1.48792	.32469
1100 BP	-18.7734	-19.9000	41	2.55266	.39866
***1000 BP**	**-15.8753**	**-15.0000**	**55**	**3.32503**	**.44835**
900 BP	-15.1843	-14.6500	124	3.37040	.30267
800 BP	-10.3303	-9.8000	262	2.40662	.14868
700 BP	-11.3602	-11.1000	300	1.95117	.11265
600 BP	-10.1082	-9.7400	153	2.43273	.19667
500 BP	-10.8633	-10.2000	33	2.06621	.35968
400 BP	-10.8665	-11.2000	71	1.70995	.20293

Table S3
Binned results for human skeletal $\delta^{13}C$ data from Mississippian and related Pre-Columbian eastern/midcontinental Native American populations including the mean, median, number of individuals samples, standard deviation and standard mean error in cal yr B.P. (present = 1950 CE). *The date at which maize consumption first averaged 50% of eastern/midcontinental Native American populations' diets based on $\delta^{13}C$ differences between diets comprised of C3 and C4 (i.e., maize) plant based protein sources[29].

UCIAMS #	Core	Drive	Depth	Material	Fraction Modern	±	$\Delta^{14}C$	±	mg C	^{14}C Age	±	Cal yr B.P.	±
132273	D-13	1	16.5	Leaf	1.2324	0.0020	232.4	2.0		-1675	15	-30	30
132275	D-13	1	62.75	Leaf	0.9853	0.0017	-14.7	1.7		120	15	110	30
142163	D-13	3	150.75	Charcoal	0.9002	0.0108	-99.8	10.8	0.021	840	100	780	100
142162	D-13	4	233.95	Charcoal	0.7996	0.0085	-200.4	8.5	0.027	1800	90	1730	90
132277	D-13	5	321.8	Stick	0.6961	0.0012	-303.9	1.2		2910	15	3040	30
142161	D-13	13	437.75	Charcoal	0.6187	0.0075	-381.3	7.5	0.035	3860	100	4270	100
132276	D-13	14	515.6	Leaf	0.5262	0.0012	-473.8	1.2	0.140	5160	20	5920	40
132274	D-13	14	573.5	Charcoal	0.4858	0.0108	-514.2	10.8	0.015	5800	180	6620	360

Table S4

Radiocarbon results from Martin Lake.
